# A pseudo‐homozygous missense variant and *Alu*‐mediated exon 5 deletion in *FARS2* causing spastic paraplegia 77

**DOI:** 10.1002/acn3.52195

**Published:** 2024-09-28

**Authors:** Shu‐Huai Lin, Jun‐Hao Xie, Jun‐Yi Jiang, Xin‐Yu Yan, Chao‐Yin Hong, Wan‐Jin Chen, Ning Wang, Xiang Lin

**Affiliations:** ^1^ Department of Neurology and Institute of Neurology of First Affiliated Hospital Institute of Neuroscience, and Fujian Key Laboratory of Molecular Neurology, Fujian Medical University Fuzhou 350005 China; ^2^ Department of Neurology National Regional Medical Center, Binhai Campus of the First Affiliated Hospital, Fujian Medical University Fuzhou 350212 China

## Abstract

*FARS2*‐associated hereditary spastic paraplegia, later onset spastic paraplegia type 77, is a rarely neurodegenerative disease. Here, we reported two affected siblings in an autosomal recessive spastic paraplegia family with a pseudo‐homozygous missense variant and *Alu*‐mediated exon 5 deletion in *FARS2*. Both patients gradually developed altered gaits and weakness in both lower limbs. In our literature review, spastic paraplegia type 77 shows high heterogeneity in clinical manifestations. Our study broadens the scope of pathogenic mechanisms of SPG77 resulting from compound heterozygous mutations in *FARS2* and provides strong evidence that deletion in *FARS2* due to recombination event mediated by Alu element.

## Introduction

Hereditary spastic paraplegia (HSP) is a group of heterogeneous diseases characterized by progressive bilateral spasticity and weakness of lower extremities.^1^ HSP is clinically classified as either pure or complicated forms, with the pure form generally limited to spasticity and weakness of lower extremities and, in some cases, associated with bladder disorders. The complicated form is accompanied by different neurological symptoms including epileptic seizure, cognitive deficits, and developmental delay, among others.^2^ The hereditary modes of HSP include autosomal dominant, autosomal recessive, X‐linked recessive, and mitochondrial inheritance.^3^ At present, more than 90 disease‐causing genes have been reported to contribute to HSP.^4^

*FARS2* encodes human mitochondrial phenylalanyl‐tRNA synthetase (Hsmt^Phe^RS), which ensures accuracy of in mitochondrial protein translation.^5^ Patients carrying loss of function variants of *FARS2* manifest as one of three distinct phenotypes: early‐onset epileptic encephalopathy, later‐onset spastic paraplegia type 77 (SPG77), and juvenile‐onset epilepsy.^6^ To date, *FARS2* mutations have been identified in 14 cases of SPG77.^7^, ^8^, ^9^, ^10^, ^11^, ^12^, ^13^, ^14^ Deletion mutations are the most common mutations besides missense mutations in SPG77 patients. The pathogenic mechanism of *FARS2* gene deletion leading to SPG77 remains unclear.

Herein, we report a pedigree with SPG77, transmitted by compound heterozygous inheritance, arising from a novel *Alu*‐mediated deletion in exon 5 of *FARS2* together with a c.1013G>A (p.A338H) missense variant. We also reviewed possible genotype–phenotype correlations between our two patients and previously reported individuals with *FARS2* deletion variants.

## Materials and Methods

All participants involved in this study from the HSP cohorts (NCT04010188) assessed at the First Affiliated Hospital of Fujian Medical University by our previously reported pipeline.^15^, ^16^ All participants underwent complete neurological examination including physical examinations, magnetic resonance imaging (MRI), electromyography (EMG), and the estimation of Spastic Paraplegia Rating Scale (SPRS),^17^ Mini–mental state examination (MMSE), and Montreal Cognitive Assessment (MoCA). This study was approved by the Ethics Committee of the First Affiliated Hospital of Fujian Medical University (No.: FYYY2006‐01‐19‐01). Written informed consents were obtained from all family members.

Genomic DNA of all participants was extracted from peripheral blood leukocytes. The causative gene in two affected siblings was explored using whole‐exome sequencing (WES) and whole‐genome sequencing (WGS). Sequencing fragments were aligned to the human genome consensus sequence (UCSC hg38). Variant calling was performed using the Genome Analysis Toolkit (GATK)^18^ and annotated with ANNOVAR.^19^ Co‐segregation of the identified *FARS2* variants was investigated by Sanger sequencing with specific primers (see Supporting Information 1: Table S1). Copy number variation (CNV) detection and calling were performed using the Sprinkle tool kit.^20^ Total RNA of all participants was extracted from peripheral blood leukocytes and were used for reverse transcription. RT‐PCR and long‐range PCR assays were performed with specific primers (see Supporting Information 1: Table S1).

## Results

### Clinical features in an autosomal recessive spastic paraplegia family

**Figure 1:**
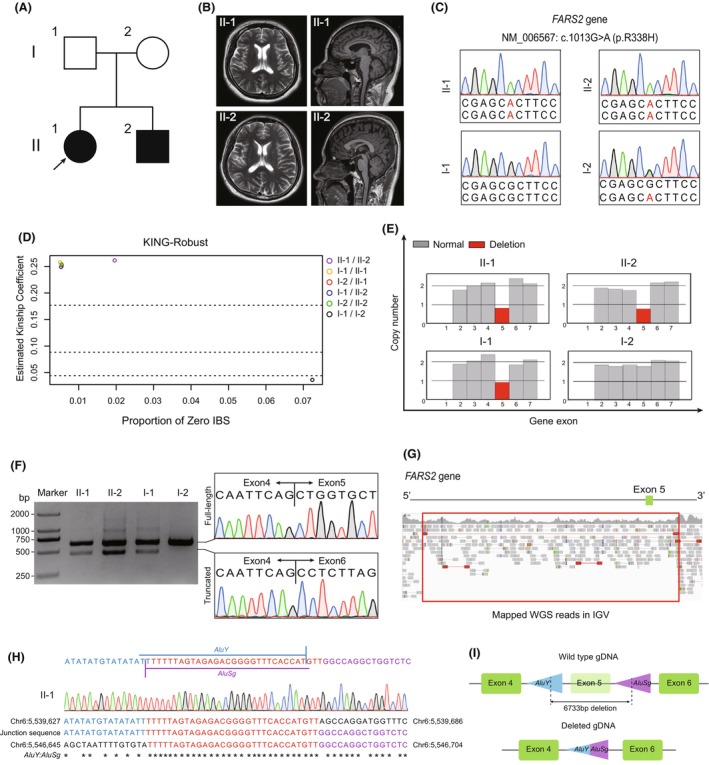
Genetic analysis of families with hereditary spastic paraplegia. (A) Family pedigree of affected siblings with suspected HSP. Filled and empty symbols represent affected and unaffected individuals, respectively. Arrow indicates the proband in this family. (B) T2‐weighted MR image showing no positive changes in II‐1 or II‐2. (C) Sanger sequencing traces of both alleles in affected individuals (top) and unaffected parents (bottom). (D) Kinship analysis by KING‐robust confirming biological paternity of I‐1 in subjects II‐1 and II‐2. (E) Copy number variation analysis of each exon for both alleles of *FARS2* in affected and unaffected family members showing the absence of paternal exon 5 in the father and both affected children. (F) Agarose gel of RT‐PCR products of *FARS2* for each family member showing a truncated allele (500 bp) in subjects I‐1, II‐1, and II‐2, but not I‐2 (left). Sanger sequencing of both bands showing two populations of mRNA, with the lower band lacking a region between exons 4 and 6 (right). (G) Whole genome sequencing data confirming the deletion at exon 5 of *FARS2*. Blank spaces indicate reduced sequencing depth and the location of gross deletion. (H) Sequencing traces for the breakpoint junction with microhomology region marked in red. Proximal reference is marked in blue; distal reference is marked in purple. (I) Diagram of the likely *Alu*‐mediated mechanism of deletion.

Two affected patients (Fig. 1A), including a woman (age 38; II‐1, the proband) and her younger brother (age 35, II‐2) gradually developed altered gaits, stiffness, and weakness in both lower limbs at 7 and 6 years old respectively, with slowly deteriorating symptoms. Proband began relying on crutches at age 27 and her younger brother at age 29. Detailed neurological examination revealed phenotypes of hypertonia, hyperreflexia, symmetrical distal muscle weakness (MRC grade 4), and positive for Babinski sign in both lower limbs. The phenotype was consistent between the affected siblings. MRI scans of the brain and spinal cord showed normal results (Fig. 1B). Electromyography was found to be normal. SPRS scores for patients II‐1 and II‐2 were 21 points and 26 points, respectively. They exhibited normal cognition and language function (MoCA scores 27 and MMSE scores 30 for patient II‐1; MoCA scores 26 and MMSE scores 29 for patient II‐2). Combined with the above clinical features, we classified both patients as pure form HSP.

### Identification of a novel missense mutation and deletion of exon 5 in *FARS2*

Whole‐exome capture identified a homozygous missense variant in *FARS2* (NM_006567: c.1013G>A: p.A338H) that was present in both patients (Fig. 1C). According to the ACMG guidelines,^21^ this variant was classified as likely pathogenic (PM1 PM2 PM3 PP1 PP3), which was described in detail in Supporting Information 1: Table S2. Interestingly, although WES suggested that the missense variant was homozygous in the patients, genotyping of the parents by Sanger sequencing indicated maternal inheritance, as the paternal *FARS2* allele carried no such variant at that position (Fig. 1C). We hypothesized three possible explanations for this phenomenon: (i) biological non‐paternity of subject I‐1, (ii) uniparental diploidy inherited from subject I‐2, or (iii) one inherited allele contained an undetected deletion. Parentage analysis by the KING‐robust estimator supported that I‐1 was indeed the biological father of II‐1 and II‐2 (Fig. 1D). Subsequent comparison of exome DNA sequence between the proband and her parents indicated that the proband carried both the maternal and paternal alleles of *FARS2*, thereby ruling out the possibility of uniparental diploidy (see Supporting Information 1: Table S3).

We then conducted CNV analysis to test the third possibility that an unknown deletion resulted in the absence of a paternal allele in exome data, which could appear as a single homozygous allele in WES data. CNV analysis indicated that the copy number of the maternal allele was indeed twice that of other family members (Fig. 1E). Sanger sequencing identified a truncation from exon 4 to exon 6 (Fig. 1F), thus confirming that subject I‐1 harbored a deletion variant that was inherited by the patients and resulted in pseudo‐homozygosity. These collective results indicated that patients II‐1 and II‐2 were, in fact, compound heterozygous and carried two distinct variants in *FARS2* that together resulted in pure form HSP.

### Deletion breakpoints contain *Alu* elements in the same orientation

WGS of the proband to detect the deletion range and breakpoints identified a 6733 bp deletion (chr19: 41896116–41941714) between intron 4 and intron 5 of *FARS2* (Fig. 1G). Sanger sequencing of the regions flanking the breakpoints revealed the presence of an *AluY* element adjacent to the breakpoint in intron 4 and an *AluSg* element at the breakpoint in intron 5 (UCSC database). Interestingly, sequence within the *Alu* elements at the breakpoints exhibited 30 bp microhomology and followed the same orientation. Further sequence analysis indicated that the boundaries of the deletion shared 81% homology with *AluY* and *AluSg* elements, respectively (Fig. 1H). This breakpoint architecture provided evidence supporting *Alu*‐mediated deletion as the likely mechanism leading to loss of exon 5 (Fig. 1I).

### Pathogenic *FARS2* deletion variants in other SPG77 cases

Only three pedigrees with SPG77 carrying deletion variants in *FARS2* have been reported to date (Table 1).^9^, ^10^, ^22^ All patients developed spastic gait, lower limb weakness, ankle clonus, and Babinski sign. One pedigree (one patient) was classified as SPG77 pure form, while the other two pedigrees (three patients) were classified as complicated form. One case in Italy was classified as the complicated form with cerebral palsy and developmental delay, while the other pedigree included two siblings who presented with dysphonia.

**Table 1:** Clinical features of the six affected individuals carrying *FARS2* variants.

	Family in this study		Family in previous study			
			Family A	Family B	Family C	
	Patient II‐1	Patient II‐2	Proband	Proband	Brother	Sister
Deletion type	ex5,chr19:41896116‐41941714	ex5,chr19:41896116‐41941714	ex.2‐4,chr6:5284218‐5442731	ex.1‐2 and ex.1 of *LYRM4*	ex.6,Chr6:5564777‐5639774	ex.6,Chr6:5564777‐5639774
Second variant	c.1013G>A, p.R338H	c.1013G>A, p.R338H	c.1082C>T, p.P361L	c.1082C>T, p.P361L	c.422G>A, p.G141E	c.422G>A, p.G141E
Sex	F	M	M	M	M	F
Age at onset	7 years	6 years	2 years	5 years	2 years	18 months
Age at evaluation	14 years	14 years	8 years	12 years	13 years	7 years
Initial symptom	Difficulty walking	Difficulty walking	DD and cerebral palsy	Gait difficulties	Delayed walking	Delayed walking
Spasticity at gait	+	+	+	+	+	+
Limb weakness (DUL/DLL)	−/+	−/+	+/+	NA	−/+	−/+
Muscle atrophy (DUL/DLL)	−/−	−/+	NA	−/+	NA	NA
Muscle tonus (DUL/DLL)	−/++	−/++	NA	−/+	NA	NA
Tremor (DUL/DLL)	−/−	−/−	−/+	−	+/+	+/+
Babinski sign	+	+	+	+	+	+
Extensor plantar response	+	+	+	+	+	+
Clonus (ankle)	+	+	+	+	+	+
Cognitive deficits	−	−	+	−	NA	NA
Epilepsy	−	−	−	−	NA	NA
Dysphonia	−	−	+	−	+	−
Developmental delay	−	−	+	−	NA	NA
Foot deformity	−	+	+	+	NA	NA
SPRS score (0–52)	21	26	NA	NA	NA	NA
SPATAX‐EUROSPA score^a^ (0–7)	5	4	NA	NA	NA	NA
MRI	Normal	Normal	NA	Normal	Normal	Normal
EEG	Normal	Normal	NA	Normal	Normal	Normal
electromyography	Normal	Normal	NA	Normal	Normal	Normal

−, absence; +, presence; DD, developmental delay; DUL/DLL, distal upper limbs/distal lower limbs; NA, not available; SPRS, Spastic Paraplegia Rating Scale.

^a^ Disability score: 0 (no functional handicap), 1 (no functional handicap but signs at examination), 2 (mild, able to run, walking unlimited), 3 (moderate, unable to run, limited walking without aid), 4 (severe, walking with one stick), 5 (walking with two sticks), 6 (unable to walk, requiring wheelchair), and 7 (confined to bed).

## Discussion

The pedigree reported here is an autosomal recessive pure form SPG77 rather than complicated form previously described in other patients with *FARS2* deletion. In our literature review, SPG77 shows high heterogeneity in clinical manifestations. Reported phenotypes are mainly characterized by a combination of spastic paraplegia, dysarthria, developmental delay, and a wide range of abnormalities in brain MRI, including diffuse brain atrophy.^23^ To establish a comprehensive perspective of genotype–phenotype correlations in SPG77, further studies, especially those focusing on pathogenic variants, will be necessary in larger HSP cohorts. Recently, *FARS2* was identified as a potential pathogenic gene to cause cardiomyopathy.^24^ However, no symptoms of myocardial damage symptoms have yet been observed in our study cohort. It will be necessary to check for cardiovascular systems in our patients during follow‐up and as a standard practice in future cases.

All previously reported deletion mutations distributed in exons 1–6 of *FARS2* could potentially compromise the structure or function of Hsmt^Phe^RS. The protein sequence contains four functional domains, including the N‐terminal region (residues 37–83), a catalytic domain (residues 84–325), a linker region (residues 326–358), and an anticodon binding domain (residues 359–451).^25^ In this current study, a deletion in exon 5, spanning residues 304–355, resulted in a partial in‐frame deletion of the aminoacylation domain and linker region. The missense variant was also situated in exon 5 and introduced a premature stop codon at R338. It is therefore reasonable to speculate that these structural changes to Hsmt^Phe^RS could interfere with its function in neurons, impairing neuronal development, which could lead to SPG77.

It is the first report of which we are aware describing an *Alu*‐mediated deletion in *FARS2* associated with SPG77. A new chimeric *Alu* element formed at the junction depending on microhomology of two *Alu* repeat elements. Greater focus on *Alu* elements is warranted in genetic analysis of HSP patients carrying deletions or duplications in future work. Notably, our initial WES analysis suggested the patients were homozygous for the missense mutation, but later found by co‐segregation analysis to be pseudo‐homozygous. It illustrates the need familial co‐segregation analysis to verify homozygous mutations.

In summary, our study broadens the scope of pathogenic mechanisms of pure form SPG77 resulting from compound heterozygous mutations at the same site in *FARS2*. These results also provide strong evidence supporting a possible role of *Alu*‐specific microhomology‐mediated recombination in the development of SPG77.

## Author Contributions

SHL designed and carried out the study. JHX drafted the manuscript and figures. JYJ coordinated the research process and helped to draft the manuscript. XYY and CYH participated in the data analysis. NW and WJC participated in clinical evaluation of the patients. XL were involved in the study design, performed clinical evaluation of the patients and critical evaluation of the manuscript.

## Conflict of Interest

The authors declare that they have no competing interests.

## Supporting information


Table S1.

## Data Availability

The data that support the findings of this study are available from the corresponding author upon reasonable request.

## References

[1] Awuah WA , Tan JK , Shkodina AD , et al. Hereditary spastic paraplegia: novel insights into the pathogenesis and management. SAGE Open Med. 2023;12:20503121231221941. 38162912 10.1177/20503121231221941 PMC10757446

[2] Meyyazhagan A , Orlacchio A . Hereditary spastic paraplegia: an update. Int J Mol Sci. 2022;23(3):1697. 35163618 10.3390/ijms23031697 PMC8835766

[3] Murala S , Nagarajan E , Bollu PC . Hereditary spastic paraplegia. Neurol Sci. 2021;42(3):883‐894. 33439395 10.1007/s10072-020-04981-7

[4] Fink JK . The hereditary spastic paraplegias. Handb Clin Neurol. 2023;196:59‐88. 37620092 10.1016/B978-0-323-98817-9.00022-3

[5] Roy H , Ling J , Alfonzo J , Ibba M . Loss of editing activity during the evolution of mitochondrial phenylalanyl‐Trna synthetase. J Biol Chem. 2005;280(46):38186‐38192. 16162501 10.1074/jbc.M508281200

[6] Yao P , Fox PL . Aminoacyl‐Trna synthetases in medicine and disease. EMBO Mol Med. 2013;5(3):332‐343. 23427196 10.1002/emmm.201100626 PMC3598075

[7] Almalki A , Alston CL , Parker A , et al. Mutation of the human mitochondrial phenylalanine‐Trna synthetase causes infantile‐onset epilepsy and cytochrome C oxidase deficiency. Biochim Biophys Acta. 2014;1842(1):56‐64. 24161539 10.1016/j.bbadis.2013.10.008 PMC3898479

[8] Barcia G , Rio M , Assouline Z , et al. Novel *Fars2* variants in patients with early onset encephalopathy with or without epilepsy associated with long survival. Eur J Hum Genet. 2021;29(3):533‐538. 33168986 10.1038/s41431-020-00757-x PMC7940479

[9] Forman EB , Gorman KM , Ennis S , King MD . *Fars2* causing complex hereditary spastic paraplegia with dysphonia: expanding the disease spectrum. J Child Neurol. 2019;34(10):621. 31106652 10.1177/0883073819846805

[10] Meszarosova AU , Seeman P , Jencik J , et al. Two types of recessive hereditary spastic paraplegia in Roma patients in compound heterozygous state; No ethnically prevalent variant found. Neurosci Lett. 2020;721:134800. 32007496 10.1016/j.neulet.2020.134800

[11] Peretz M , Tworowski D , Kartvelishvili E , Livingston J , Chrzanowska‐Lightowlers Z , Safro M . Breaking a single hydrogen bond in the mitochondrial tRNAPhe ‐PheRS complex leads to phenotypic pleiotropy of human disease. FEBS J. 2020;287(17):3814‐3826. 32115907 10.1111/febs.15268 PMC7540514

[12] Sahai SK , Steiner RE , Au MG , et al. *Fars2* mutations presenting with pure spastic paraplegia and lesions of the dentate nuclei. Ann Clin Transl Neurol. 2018;5(9):1128‐1133. 30250868 10.1002/acn3.598 PMC6144452

[13] Vantroys E , Larson A , Friederich M , et al. New insights into the phenotype of *Fars2* deficiency. Mol Genet Metab. 2017;122(4):172‐181. 29126765 10.1016/j.ymgme.2017.10.004 PMC5734183

[14] Yang Y , Liu W , Fang Z , et al. A newly identified missense mutation in *Fars2* causes autosomal‐recessive spastic paraplegia. Hum Mutat. 2016;37(2):165‐169. 26553276 10.1002/humu.22930

[15] Qiu YS , Zeng YH , Yuan RY , et al. Chinese patients with hereditary spastic paraplegias (HSPs): a protocol for a hospital‐based cohort study. BMJ Open. 2022;12(1):e054011. 10.1136/bmjopen-2021-054011 PMC8753405 35017251

[16] Dong EL , Wang C , Wu S , et al. Clinical spectrum and genetic landscape for hereditary spastic paraplegias in China. Mol Neurodegener. 2018;13(1):36. 29980238 10.1186/s13024-018-0269-1 PMC6035405

[17] Schüle R , Holland‐Letz T , Klimpe S , et al. The Spastic Paraplegia Rating Scale (SPRS): a reliable and valid measure of disease severity. Neurology. 2006;67(3):430‐434. 16894103 10.1212/01.wnl.0000228242.53336.90

[18] McKenna A , Hanna M , Banks E , et al. The genome analysis toolkit: a Mapreduce framework for analyzing next‐generation DNA sequencing data. Genome Res. 2010;20(9):1297‐1303. 20644199 10.1101/gr.107524.110 PMC2928508

[19] Wang K , Li M , Hakonarson HH . Annovar: functional annotation of genetic variants from high‐throughput sequencing data. Nucleic Acids Res. 2010;38(16):e164. 20601685 10.1093/nar/gkq603 PMC2938201

[20] Fromer M , Purcell SM . Using XHMM software to detect copy number variation in whole‐exome sequencing data. Curr Protoc Hum Genet. 2014;81:7.23.1‐7.23.21. 10.1002/0471142905.hg0723s81 PMC4065038 24763994

[21] Richards S , Aziz N , Bale S , et al. ACMG laboratory quality assurance committee. Standards and guidelines for the interpretation of sequence variants: a joint consensus recommendation of the American College of Medical Genetics and Genomics and the Association for Molecular Pathology. Genet Med. 2015;17(5):405‐424. 25741868 10.1038/gim.2015.30 PMC4544753

[22] Panzeri E , Citterio A , Martinuzzi A , Ancona V , Martini E , Bassi MT . Case report: a novel *Fars2* deletion and a missense variant in a child with complicated, rapidly progressive spastic paraplegia. Front Genet. 2023;14:1130687. 37152989 10.3389/fgene.2023.1130687 PMC10154595

[23] Zhang X , Xiang F , Li D , Yang F , Yu S , Wang X . Adult‐onset combined oxidative phosphorylation deficiency type 14 manifests as epileptic status: a new phenotype and literature review. BMC Neurol. 2024;24(1):15. 38166857 10.1186/s12883-023-03480-4 PMC10759640

[24] Li B , Liu F , Chen X , et al. *Fars2* deficiency causes cardiomyopathy by disrupting mitochondrial homeostasis and the mitochondrial quality control system. Circulation. 2024;149(16):1268‐1284. 38362779 10.1161/CIRCULATIONAHA.123.064489 PMC11017836

[25] Klipcan L , Levin I , Kessler N , Moor N , Finarov I , Safro M . The Trna‐induced conformational activation of human mitochondrial phenylalanyl‐Trna synthetase. Structure. 2008;16(7):1095‐1104. 18611382 10.1016/j.str.2008.03.020

