# Supplementary material for: A pseudo‐homozygous missense variant and Alu‐mediated exon 5 deletion in FARS2 causing spastic paraplegia 77
Source: Ann Clin Transl Neurol. 2024 Sep 28;11(11):3019–24. doi: 10.1002/acn3.52195 (PMC11572740; doi:10.1002/acn3.52195)
Supplement: Supplementary file 1 — Table S1. [file ACN3-11-3019-s001.docx]

**Supplementary Information**

**Table S1. Primer sequences used in this study.**

| Name/purpose | Forward sequence (5’→3’) | Reverse sequence (5’→3’) |
| --- | --- | --- |
| Sanger sequencing of the human *FARS2* gene | | |
| *FARS2-c.1013G>A* | TGGAGATGAGAAAGCGTGGAAG | TCAAGTACATGATCCTGTCG |
| Reverse transcription PCR (RT-PCR) targeting the exon 5 deletion in *FARS2* | | |
| RT-PCR | CCAAGCATGAGTTATTTGCTGG | GTCTTGTGCGTCTTTGGATG |
| Long-range PCR targeting the deletion breakpoint in *FARS2* | | |
| Long-range PCR | GACAGGAGTCTCGCTCTGTG | CATCAGAGTTCTATGGCATAGG |

**Table S2. Summary of features for the novel missense variant c.1013G>A in the *FARS2* gene.**

| Inheritance | Zygosity | AA change | Location | ExAC-EAS | gnomAD-EAS | 1KGP | WBBC | PolyPhen-2 | MutationTaster | CADD | GERP^++^ |
| --- | --- | --- | --- | --- | --- | --- | --- | --- | --- | --- | --- |
| Maternal | Complex heterozygote | p.A338H | Linker region domain | 0 | 0 | 0 | 0 | Probably_ damaging | Disease_causing | 26.1 | 4.39 |

AA, amino acid; EAS, East Asian

**Table S3. Summary of WES analysis for the *FARS2* alleles of all family members.**

|  | | II-1 | | II-2 | | I-1 | | I-2 | |
| --- | --- | --- | --- | --- | --- | --- | --- | --- | --- |
| CHROM | POS | Allele1 | Allele2 | Allele1 | Allele2 | Allele1 | Allele2 | Allele1 | Allele2 |
| 6 | 5369322 | G | C | G | C | G | G | G | C |
| 6 | 5431340 | A | G | A | G | A | A | A | G |
| 6 | 5431960 | G | A | G | A | A | G | A | A |
| 6 | 5545521 | - | A | - | A | G | - | G | A |
| 6 | 5609536 | A | T | A | T | A | A | A | T |
| 6 | 5609621 | G | T | G | T | T | G | T | T |
| 6 | 5609691 | G | A | G | A | A | G | G | A |
